# Analysis of Static and Cyclic Properties of 316L and AlSi10Mg in Conventional Casting and Additive Manufacturing

**DOI:** 10.3390/ma17235861

**Published:** 2024-11-29

**Authors:** Vladimír Chmelko, Matúš Margetin, Ivana Zetková, Martin Norek, Filip Likavčan

**Affiliations:** 1 Institude of Applied Mechanics and Mechatronics, Slovak University of Technology, Námestie Slobody 17, 81231 Bratislava, Slovakia; matus.margetin@stuba.sk (M.M.); martin.norek@stuba.sk (M.N.); filip.likavcan@stuba.sk (F.L.); 2 Regional Technological Institute, Faculty of Mechanical Engineering, University of West Bohemia, Univerzitni 22, 306 14 Pilsen, Czech Republic; zetkova@fst.zcu.cz

**Keywords:** additive manufacturing, aluminium alloy, fatigue properties, 316L, tension and torsion cyclic tests

## Abstract

The paper presents the original results of cyclic testing of materials that are identical in chemical composition but produced by two different technologies: conventional metallurgy and additive manufacturing. For the aluminium alloy AlSi10Mg and the austenitic steel 316L, tensile curves, tension–compression and torsion alternating fatigue curves are experimentally obtained and presented. The experimental results are compared for two fabrication technologies—conventional metallurgy and additive DLMS technology. The results indicate a significant effect of anisotropy on the fatigue performance of the AM materials and a different slope of the fatigue life curves in the cyclic torsion versus cyclic tension–compression. The static and, in particular, the fatigue properties of both materials are discussed in relation to the microstructure of the materials after conventional production and after additive manufacturing. This comparison allowed us to explain both the causes of the anisotropy of the AM materials and the different slope of the curves for normal and shear stresses under cyclic loading. Using the example of the strength assessment of bicycle frames, the possibility of progressively wider use of additive manufacturing for load-bearing structures is presented.

## 1. Introduction

**Figure 1:**
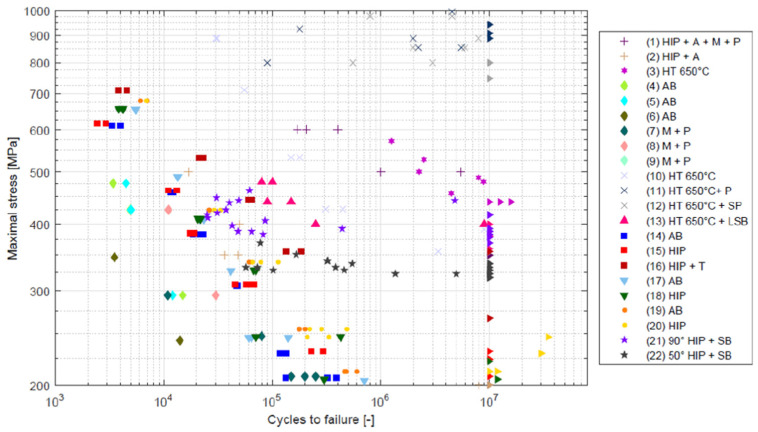
Large scatter of results of S-N curves of TI-6Al-4 alloy produced by DLMS technology [3].

Over the last decade, additive manufacturing of metals has been the subject of wide research interest in the field of their mechanical properties and potential applications in the operation of structures [1,2]. This is the usual intermediate step between the appearance of new technologies and materials and their implementation in engineering practice. During this decade, many experimental results documenting the mechanical properties of additively manufactured metallic materials have been accumulated. The research in this field to date has revealed a great variability in the properties of metals produced by additive technologies, as documented by the S-N curve diagram of the Ti-6Al-4V alloy (Figure 1) published in [3]. Among the existing additive manufacturing technologies for metallic materials (an overview of different additive manufacturing technologies can be found, for example, in [4,5]), DLMS and SLM appear to be the most suitable technologies for stress-containing components under service conditions of structures. Other technologies such as binder jetting, FFM and FDM have lower requirements and costs but are not suitable for stressed elements in structures in terms of strength and cyclic properties. In general, additive manufacturing, even with local remelting of the input powder material, leads to differences in mechanical properties compared to conventional metallurgy. Strength properties such as Re, Rm and ductility change to lower values. In the case of the beneficial effect of rapid cooling on the microstructure of the material, they may also increase [5]. Even the Young’s modulus is not always unaffected by additive technology—it only changes to lower values in the case of a change [5,6]. The influence of additive manufacturing on the fatigue properties of materials is unambiguous—always negative. An unintended reality of the current state of additive technologies is the variance in the mechanical properties of materials even within the same type of additive technology. The application of even today’s most widely used DLMS technology by different manufacturers gives a large scatter in the results of cyclic tests, as documented by the comparison shown in Figure 1.

Despite this state of affairs, the process of the standardisation of additive manufacturing and its parameters will continue and additively manufactured components will be more and more frequently used in engineering practice. From the results accumulated so far, it is clear that even the current level of additive manufacturing technology allows the use of such manufactured components for static and quasi-static loading. The problem of additively manufactured components, apart from the surface quality in the as-built condition, is so far mainly in the area of fatigue properties [7,8,9,10,11,12,13,14]. In this paper, two metal alloys, AlSi10Mg and 316L (and partly steel MS1), will be given special attention. Both alloys are very widely used in practice even with conventional manufacturing technology and for both materials specimens of identical chemical composition were obtained without additional heat treatment. A comparison of both static strength characteristics and their cyclic properties in alternating tension–compression and in torsion will help their deployment in practice in the operating conditions of real structures additively manufactured. Attention will be paid to the causes of the differences in their properties after conventional production compared to additive manufacturing. The difference in the use of these two technologies in design will be documented on a bicycle frame structure.

## 2. Materials

In this study, two materials, AlSi10Mg and 316L, will be analysed in more detail. For both materials, measurements of strength and fatigue properties were carried out on chemically identical alloys produced by two different technologies: conventional metallurgy and DLMS technology. The additive manufacturing parameters of the test specimens of both materials were set according to the manufacturer’s standards (EOS) and have not been modified.

The AlSi10Mg alloy is a eutectic Al and Si alloy favoured by design engineers, with applications in the construction of internal combustion engines, wheel rims, bicycle frames and aerospace components. The chemical composition is documented in Table 1.

The properties of this alloy, except for its cyclic properties in torsion, are reported in the literature [15,16,17,18,19].

Austenitic steel 316L is a basic stainless steel with wide applications in transportation infrastructure, medicine and other fields [20,21,22]. The chemical composition of this austenitic steel is documented in Table 2.

**Figure 2:**
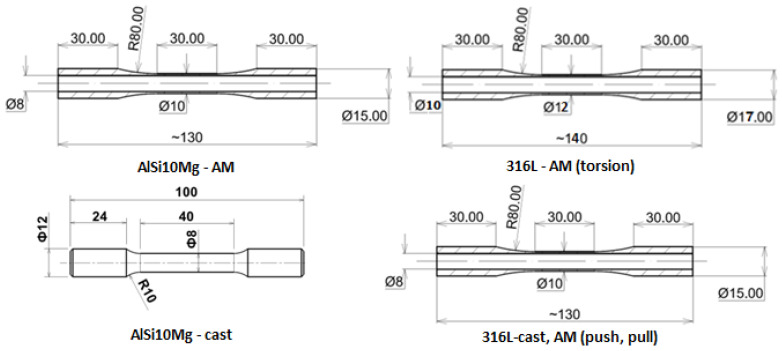
Specimens of materials used for static and cyclic tests (dimensions are in mm).

Only cylindrical specimens were used for the cyclic tests. The shape of the specimens used for the cyclic tests is shown in Figure 2.

## 3. Results

**Figure 3:**
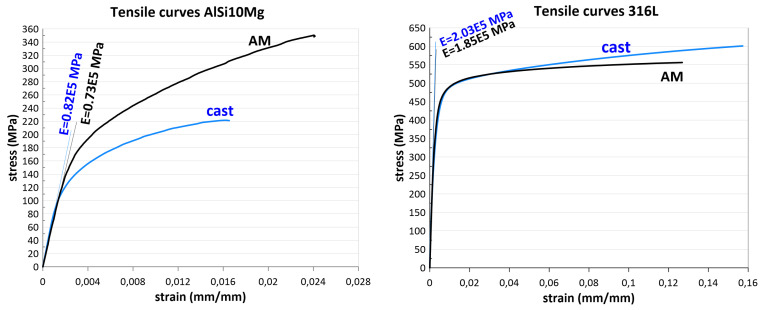
Comparison of tensile curves between additive manufacturing and conventional metallurgy.

The effect of additive manufacturing on the strength properties of metals is not conclusive. AlSi10Mg aluminium alloy has significantly higher yield and ultimate strengths after additive manufacturing compared to conventional metallurgy. In contrast, the Young’s modulus value decreased due to additive manufacturing. The properties of 316L austenitic steel are affected inversely by additive manufacturing—the strength of the steel after conventional manufacturing is higher than that of AM. For both materials, additive manufacturing caused a decrease in the Young’s modulus value (Figure 3). However, a 12% and 10% decrease in AlSi10Mg and 316L, respectively, will not limit the use of these materials from a strength point of view, since the yield strength is the key parameter for the strength assessment of structural elements.

A negative consequence of additive manufacturing on material properties is anisotropy, which is the result of the influence of the direction of deposition of the material layers [22,23,24,25]. This effect has been investigated in work [15] on aluminium alloy; the results are summarised in Table 3. Concerning the change in strength properties depending on the direction of material addition, it can be concluded that anisotropy is a reality and is measurable in technologies that locally melt powder material. The degree of anisotropy is not significant in terms of strength dimensioning. On the contrary, the anisotropy due to additive manufacturing is more significant in the Young’s modulus. Its variations depending on the direction of material addition in some materials have to be considered in numerical simulations (for such simulations that require deformation accuracy) [25], which increases the computational time consumption of the simulations.

**Figure 4:**
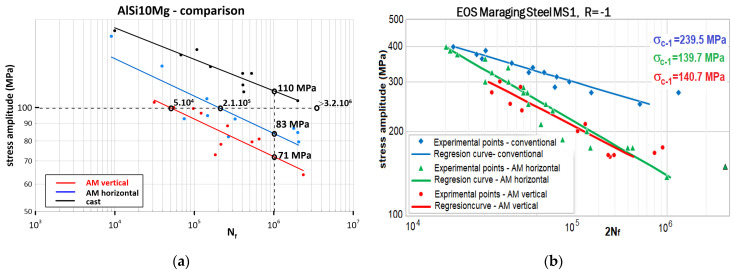
Effect of material anisotropy due to additive manufacturing on the cyclic properties of AlSi10Mg and MS1 (1.2709). (**a**) Basquin curves forAlSi10Mg; (**b**) Basquin curves for 316L.

The anisotropy is more significant in the cyclic properties of AM materials. This phenomenon is analysed in more detail in [23,24,25,26,27]. For a stress amplitude level of 100 MPa, the anisotropy due to the AM of alloy AlSi10Mg causes several orders of magnitude difference in fatigue lifetime compared to conventional manufacturing. For a fatigue life length of 10^6^ cycles, AM anisotropy affects the allowable stress amplitude by 17%, compared to conventional manufacturing, where the difference in the magnitude of the allowable amplitude is up to 1.55 times, as documented in Figure 4a.

**Figure 5:**
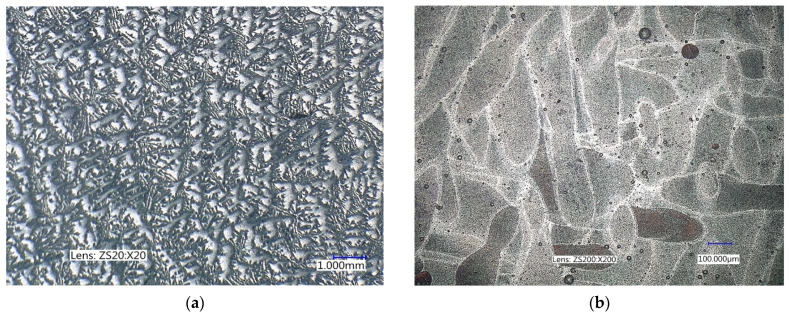
Comparison of the microstructure of conventionally cast AlSi10Mg with the microstructure after additive manufacturing. (**a**) conventionally cast; (**b**) additively manufacturing.

To understand the causes of material anisotropy stemming from AM, Figure 4b is presented with the cyclic test results for MS1 steel (1.2709). The effect of the deposition direction of the layers on the anisotropy of the fatigue properties is significantly less compared to AlSi10Mg. The explanation for this difference is hidden in the microstructure combined with the fatigue damage mechanism of the material (this line of thinking has been followed recently by more and more authors [28,29,30,31,32]). On the one hand, the AlSi10Mg material is a eutectic alloy of Al and Si in which intermetallic Mg2Si particles are formed during crystallisation (see Figure 5a). These solidify the underlying eutectic matrix and are obstacles to the movement of the slip planes. In additive manufacturing, due to the high cooling rate, these intermetallic particles do not form. The matrix formed by Al and Si solid solution is practically homogeneous and the only discontinuities are defects caused by imperfections in the additive technology (lack of fusion, porosity, etc.)—see Figure 5b.

**Figure 6:**
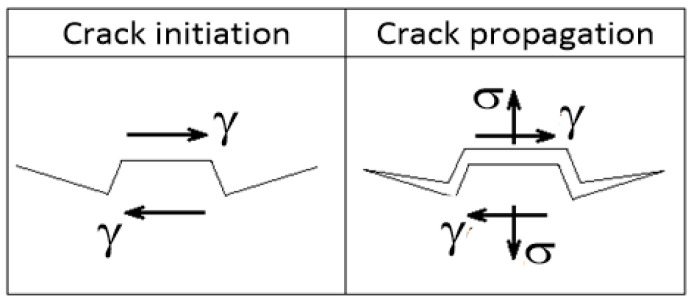
Shear strain is critical to the initiation of fatigue crack in the microstructure of the material, and the combination of shear strain and normal stress is crucial in the process of its propagation.

These defects are orientated in accordance with the direction of application of the layers. Under cyclic loading, defects orientated in the planes of maximum shear stress are accelerators of fatigue damage progression. As a result, the direction of deposition of the material layers that produces defects orientated closest to the planes of the maximum shear stress under cyclic loading will result in shorter cycles to fracture. Perpendicular to this direction of deposition of material layers, fatigue lives will be significantly longer (Figure 4 AlSi_10_Mg). This finding is consistent with the Fatemi–Socie criterion model formulated for multiaxial fatigue [33] (Figure 6).

**Figure 7:**
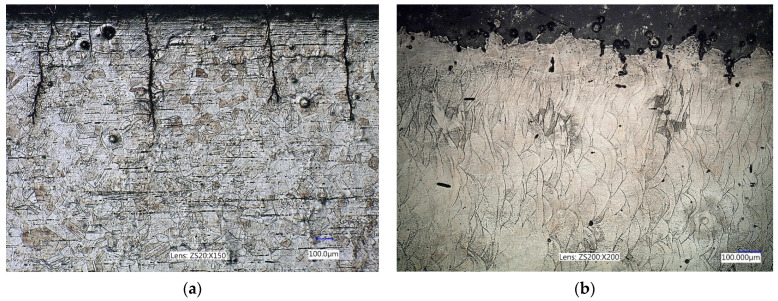
Comparison of the microstructure of conventionally cast and rolled 316L steel (**a**) with the microstructure after additive manufacturing (**b**).

On the other hand, MS1 is a steel with a high content of alloying elements such as Ni, Co, Mo, etc. These elements form many intermetallic particles that constitute barriers to slip processes in the base matrix (Figure 7). This generally guarantees a higher fatigue strength of the material (upper curve in Figure 4b), because the microstructure creates barriers to the propagation of microcracks in all directions. In the case of additive manufacturing, the dense network of barriers to microcrack movement reduces the influence of the orientation of internal defects caused by the direction of deposition of the material layers on the propagation speed of these microcracks (bottom two curves in Figure 4b).

The effect of additive manufacturing on the cyclic properties of metallic materials is investigated to assess the applicability of this manufacturing technology to structural elements subjected to variable loading. This is a common reality in the field of structural service and a major challenge for the development of additive manufacturing technologies.

Cyclic testing of material specimens in alternating tension–compression as well as torsion was carried out using an MTS 370.02 electrohydraulic frame pulsator. The frequencies were designed to avoid overheating of the samples. The 316L steel samples were particularly susceptible to this due to their high ductility and deformability conditioned by the area-centred crystalline austenite grid.

**Figure 8:**
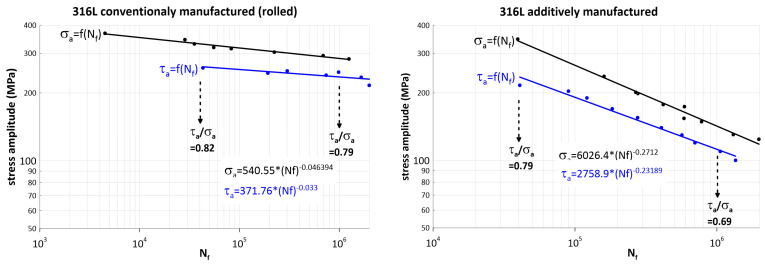
Fatigue curves for tension and torsion of 316L steel produced conventionally (**left**) and additively (**right**).

The cyclic test results for 316L austenitic steel are shown in Figure 8. In the region of high-stress amplitudes (near-low cycle fatigue), the fatigue lifetimes of conventionally produced and additively produced steel are practically comparable. The different fatigue properties start to become apparent as the number of cycles in the so-called high-cycle region of the fatigue life increases.

For 10^6^ cycles, the average allowable amplitude for conventionally fabricated steel in torsion is 235.65 MPa versus 285.75 MPa for tension–compression, giving a ratio of 0.82. For 4.10^4^ cycles, the average allowable amplitude for AM in torsion is 262 MPa versus 330.6 MPa for tension-compression, giving a ratio of 0.79.

For 10^6^ cycles, the average allowable amplitude for AM in torsion is 112 MPa versus 142 MPa for tension–compression, giving a ratio of 0.78. For 4.10^4^ cycles, the average allowable amplitude for AM in torsion is 236.35 MPa versus 340.4 MPa for tension–compression, giving a ratio of 0.69.

It can be seen from the experimental results that the ratio of the cyclic strength of the material for shear stresses to the cyclic strength for normal stresses is quite high, practically constant for conventional manufacturing, and for AM at longer lifetimes the ratio of the shear amplitude to the normal stress amplitude increases slightly. The reduction in fatigue strength due to additive manufacturing technology for 10^6^ cycles is twofold for normal stresses and 2.1 times for shear stresses. In the fatigue life cycle number region of 4.10^4^, the fatigue strength (cycle allowable amplitude) in tension and torsion was comparable for both manufacturing technologies.

These results are a reflection of the overall composition and microstructure of the steel under investigation, which is overall suitable for production by powder metallurgy. The imperfection of additive manufacturing, which produces defects in the microstructure, causes them to have a strong influence on the reduction in the fatigue strength significantly only in the high-cycle region.

**Figure 9:**
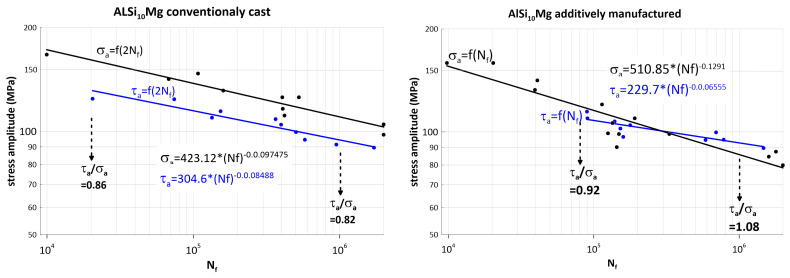
Fatigue curves for tension and torsion of AlSI10Mg aluminium alloy produced conventionally (**left**) and additively (**right**).

The cyclic test results for aluminium alloy AlSI10Mg are shown in Figure 9. The trend of decreasing fatigue strength with the increasing number of cycles is also evident, but is significantly less marked compared to 316L steel.

For 10^6^ cycles, the average allowable torsional amplitude for the conventionally produced aluminium alloy is 94.3 MPa versus 110 MPa for tension–compression, which is a ratio of 0.86. For 2.10^4^ cycles, the average allowable amplitude for torsion is 131.4 MPa versus 161 MPa for tension–pressure, giving a ratio of 0.82.

For a service life of 10^6^ cycles, the average allowable amplitude for AM in torsion is 92.86 MPa versus 85.84 MPa for tension–pressure, giving a ratio of 1.08. For a service life of 8.10^4^ cycles, the average allowable amplitude for AM in torsion is 109.6 MPa versus 119 MPa for tension–pressure, giving a ratio of 0.92.

From the experimental results, it can be seen that the ratio of the fatigue strength of the material for shear stresses to the fatigue strength for normal stresses is high, higher than that of 316L steel, and is practically constant for conventional production as well.

However, a surprising anomaly occurs in additive manufacturing—the fatigue strength in alternating torsion exceeds the tensile-compressive fatigue strength. This phenomenon is very rare in conventionally produced metals [34], but occurs frequently in additive manufacturing. The reduction in fatigue strength for 10^6^ cycles is 1.3-fold for normal stresses (depending on the direction of material addition, this can be as much as 1.55-fold—see Figure 3); for shear stresses, the fatigue strength is virtually unaffected by additive manufacturing. This phenomenon can probably be attributed to the different mode of crack propagation in the materials [35,36,37]. The most commonly occurring mode I is a reflection of the high sensitivity of the crack (defect) to normal stresses, and in the case of the parallelism of the σ_a_ = f(N_f_) and τ_a_ = f(N_f_) curves, the materials are similarly sensitive to non-planar shear stresses (represented by torsion), which is mode III—this could be the case for both 316L steel and the AlSI10Mg aluminium alloy in the conventional casting version. Additively manufactured AlSi10Mg is probably less susceptible to crack (defect) propagation in the non-planar shear stress (torsion) mode. The degree of reduction in fatigue strength in the high-cycle fatigue region is probably related to the orientation of the defects formed in a particular material addition direction.

The different behaviour of the aluminium alloy under cyclic loading compared to additively manufactured 316L steel is also due to the different microstructure of the material—AlSi10Mg is a eutectic alloy of Al and Si, and in the non-heat-treated state it is a homogeneous solid solution with no reinforcing particles and no impediments to the propagation of microcracks [38,39].

## 4. Discussion: Effectiveness of the Use of Additive Technology in the Manufacture of Bicycle Frames

The specific feature of aluminium alloy bicycle frames is the combination of several production technologies: gravity casting, die casting, hydroforming and welding. The identical chemical composition of the material after different technologies for the production of structural components significantly changes the mechanical properties of the aluminium alloys [40,41]. Table 4 documents the strength properties of AlSi7Mg alloy as a result of different technological processes of final component production. Additionally, the individual parts of the bicycle frame are joined by welding, which, by thermal overheating, will significantly reduce the properties at the weld node of a material that has had enhanced mechanical properties by previous heat treatment.

**Figure 10:**
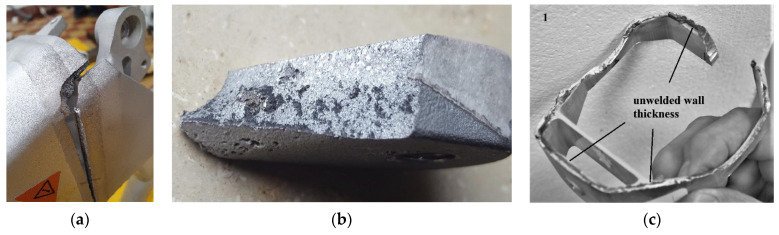
Defects in welds and castings of bicycle frame components may also be highlighted: (**a**) fatigue fracture in the middle of the weld cover; (**b**) voids in the metal structure after casting; (**c**) weld imperforations in variable wall thickness.

The complex shapes involved in the need to accommodate new e-bike components often make it impossible for designers to avoid using technologies such as casting or welding. The mechanical properties of the resulting frame are therefore not homogeneous throughout the structure. For the strength assessment of the frame, it is therefore necessary to take into account the properties attributable to the individual components. Voids in castings and welds of complex shape not welding with varying wall thickness around the perimeter are facts that significantly reduce the fatigue strength and service life of bicycle frames, as documented in Figure 10b,c.

**Figure 11:**
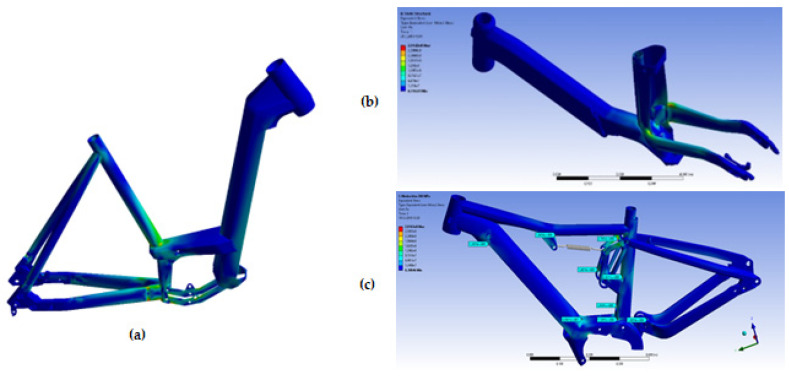
Typical cases of electric bicycle frames with the highest stress locations.

These causes could lead to an opening of space for the effective use of additive manufacturing. The procedure for calculating the fatigue life of a frame in its design is methodically described in [41]. From the stress–-strain analyses for different loading conditions of different types of bicycle frames documented in Figure 11, it can be seen that most of the locations with the largest stress values are the connection points of the different structural parts. The joints are most commonly welded and the welds are also the locations of the most frequent fatigue fracture failures when the frames are tested on the test stands (Figure 10a).

From the aluminium alloy AlSi10Mg, the first bicycle frame was additively manufactured in one piece without the need for welding or any other material joining technology (2019, [42]). What was then primarily a marketing initiative is now much closer to an engineering realisation. In terms of the necessary static strength, the frame has no limitations; the yield strength of the additively manufactured material is higher than in the case of conventional casting (Figure 3). The maximum allowable stresses in terms of cyclic loading have to be derived from loading scenarios that are prescribed by experimental testing of frames in accredited laboratories. In particular, the highest stress values arise in two loading scenarios—the combination of vertical and horizontal forces on the pedals (representing travel over bumps and obstacles) and the horizontal force acting on the front fork (representing travel over an elevated obstacle).

The load cycles of bicycle frames represent an approximately vanishing cycle R ≅ 0, i.e., a cycle with a significant mean value. In [39], S-N curves are presented for different types of welded joints of Al6061 alloy plates, which is closely related in properties to AlSi10Mg alloy. The allowable stress decay cycles for a lifetime of 10^5^ cycles are in the range 70–140 MPa, for a lifetime of 10^6^ cycles from 50–100 MPa. For the detail of the butt weld of the plates, which is the closest of the above joints to the butt welds of the tubes on the bicycle frame, the permissible stress ranges are 100 MP for a life of 10^5^ cycles and 70 MPa for a life of 10^6^ cycles to fatigue fracture.

These values are comparable to, and sometimes even lower than, the allowable alternating cycle amplitudes of additively manufactured AlSi10Mg alloy—from the graph in Figure 4 it is possible to identify allowable alternating cycle amplitudes from 92 to 105 MPa for a life of 10^5^ cycles and from 71 to 83 MPa for 10^6^ cycles to fatigue fracture. The range of allowable amplitudes is determined by the direction of material addition. Given these values of stress amplitudes, it is clear that a frame fabricated as a single unit by additive manufacturing would have a comparable fatigue life to a frame made from a conventionally fabricated aluminium alloy. The reason for this is the significant decrease in the allowable stress amplitudes and strains at the welded joints. The advantage of additive manufacturing would be the elimination of defective unwelded joints, as shown in Figure 10.

It appears that despite the significant reduction in cyclic properties of materials due to additive manufacturing, their first applications even in structures requiring sizing for the high-cycle fatigue life region are beginning to be justified in engineering terms.

## 5. Conclusions

The key difference between conventional metallurgy and additive manufacturing is in the cooling rate and in the presence of defects from which the final microstructure results. This is then reflected in strength but especially in fatigue properties. The continuous development of additive manufacturing technologies for metallic materials brings them closer to applications for cyclically loaded load-bearing elements of structures. In particular, the necessary standardisation of production parameters to guarantee a minimum level of cyclic properties is a major challenge. Another challenge is the refinement of additive manufacturing in order to reduce the anisotropy of the properties of the materials produced in this way. One possible avenue for developing this technology to reduce the influence of anisotropy on fatigue performance could be to adapt the direction of layer addition to the locations with the highest variable amplitudes in the design operation.

In this paper, the results of the measurements of the static and cyclic properties of two different metallic materials—aluminium alloy AlSI10Mg and austenitic steel 316L—are presented. Both materials are compared in the form of conventional production with the as-built condition after DLMS additive manufacturing technology. On the basis of the results obtained, the following conclusions can be formulated:

1. Practically all the strength characteristics of the metallic materials acquire anisotropy due to the direction of the material addition, the most significant being the Young’s modulus value;
2. The effect of additive manufacturing on the strength properties can be both positive and negative; Young’s modulus due to additive manufacturing always decreases due to defects that weaken the cross-section;
3. A significant reduction in the fatigue strength of additively manufactured metals is manifested particularly in the high-cycle region, with the degree of reduction depending on the composition of the microstructure and the susceptibility of the material to the relevant mode of crack propagation;
4. Anisotropy of cyclic properties is an inevitable consequence of current AM technologies, the degree of its negative manifestation depending on the microstructure composition of the particular material.

Despite all the negative effects of additive technologies on the fatigue properties of metals, DLMS or SML technology has the potential to be used in the industrial series production of complete structures that require joining by welding in conventional manufacturing technologies (e.g., bicycle frames).

## Figures and Tables

**Table 1:** Chemical composition of AlSi10Mg.

AlSi10Mg	Si (%)	Mg (%)	Fe (%)	Ti (%)	Mn (%)	Cu (%)	Zn (%)	Cr (%)
Conventional metallurgy	10.20	0.346	0.112	0.121	0.046	0.0017	0.02	0.002
Additive manufacturing	10.1	0.38	0.09	<0.03	<0.03	<0.03	<0.03	-

**Table 2:** Chemical composition of 316L.

316L	Si (%)	Mg (%)	Fe (%)	Ti (%)	Mn (%)	Cu (%)	Zn (%)	Cr (%)
Conventional metallurgy	10.20	0.346	0.112	0.121	0.046	0.0017	0.02	0.002
Additive manufacturing	10.1	0.38	0.09	<0.03	<0.03	<0.03	<0.03	-

**Table 3:** Anisotropy of strength properties of AlSi10Mg (is in accordance with [15]).

AlSi_10_Mg	45°	90°	180°
Ultimate stress (MPa)	360–370 (367)	325–390 (366)	316–350 (336)
Yield stress (MPa)	230–268 (252)	220–260 (248)	224–250 (236)
Ductility (%)	6.0–9.6 (8.2)	5.6–10.0 (7.63)	10.0–16.3 (12.83)

**Table 4:** Strength properties of AL alloys for different production technologies.

Technology	Re (MPa)	Rm (MPa)	A
Low-pressure casting AlSi7Mg0.3	200	240	5%
Pressure casting AlSi7Mg0.3	260	320	8%
Forging AlSi7Mg0.3	300	340	10%
A6061 T4	145	240	-
A6061 T6	276	310	-

## Data Availability

Dataset available on request from the authors.
